# Incompatibility and Interchangeability in Molecular Evolution

**DOI:** 10.1093/gbe/evac184

**Published:** 2022-12-30

**Authors:** Daniel B Sloan, Jessica M Warren, Alissa M Williams, Shady A Kuster, Evan S Forsythe

**Affiliations:** Department of Biology, Colorado State University, Fort Collins, Colorado; Center for Mechanisms of Evolution, Biodesign Institute and School of Life Sciences, Arizona State University, Tempe, Arizona; Department of Biological Sciences, Vanderbilt University, Nashville, Tennessee; Department of Biology, Colorado State University, Fort Collins, Colorado; Department of Biology, Colorado State University, Fort Collins, Colorado

**Keywords:** cytonuclear, epistasis, horizontal gene transfer, hybridization, protein–protein interactions

## Abstract

There is remarkable variation in the rate at which genetic incompatibilities in molecular interactions accumulate. In some cases, minor changes—even single-nucleotide substitutions—create major incompatibilities when hybridization forces new variants to function in a novel genetic background from an isolated population. In other cases, genes or even entire functional pathways can be horizontally transferred between anciently divergent evolutionary lineages that span the tree of life with little evidence of incompatibilities. In this review, we explore whether there are general principles that can explain why certain genes are prone to incompatibilities while others maintain interchangeability. We summarize evidence pointing to four genetic features that may contribute to greater resistance to functional replacement: (1) function in multisubunit enzyme complexes and protein–protein interactions, (2) sensitivity to changes in gene dosage, (3) rapid rate of sequence evolution, and (4) overall importance to cell viability, which creates sensitivity to small perturbations in molecular function. We discuss the relative levels of support for these different hypotheses and lay out future directions that may help explain the striking contrasts in patterns of incompatibility and interchangeability throughout the history of molecular evolution.

**Significance**

As gene sequences diverge, molecular interactions between proteins can be disrupted, resulting in harmful functional consequences. For some genes, these incompatibilities arise rapidly, contributing to the early stages of speciation. In other cases, they are slow to emerge, and interchangeability can be maintained for billions of years, as evidenced by examples of horizontal gene transfer and functional replacement events between highly divergent evolutionary lineages. This review explores explanations for why different types of molecular interactions follow these contrasting evolutionary paths that range from incompatibility to interchangeability.

## Introduction

**Fig. 1.**
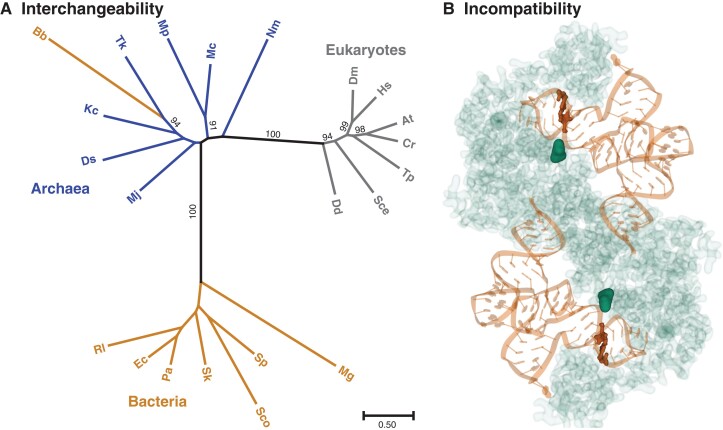
The paradox of interchangeability and incompatibility illustrated with aaRS genes: (*A*) An example of interchangeability between anciently divergent copies of phenylalanine aaRS via HGT from archaea to the bacterial lineage that includes spirochaetes, represented here by *Borrelia burgdorferi* (Bb; Woese et al. 2000). Amino-acid sequences for phenylalanine aaRS orthologs were recovered with SHOOT (Emms and Kelly 2022) using *B. burgdorferi* (ADQ30774) as a query sequence, aligned with MAFFT (Katoh and Standley 2013), and used for maximum-likelihood phylogenetic inference with IQ-TREE (Minh et al. 2020). Bipartitions with >90% support from ultrafast bootstrap pseudoreplicates are indicated. Aligned sequences with full taxon names are provided as supplemental material (supplementary File S1, Supplementary Material online). (*B*) A contrasting example of aaRS-tRNA incompatibility based on only a single-nucleotide substitution in the tRNA and a single amino-acid substitution in the aaRS. The structural model represents a tyrosine aaRS dimer (green) complexed with two tRNA-Tyr molecules (orange). The highlighted residues and base pairs indicate the positions that are homologous to sites where substitutions occurred in *Drosophila*, leading to an incompatibility (Meiklejohn et al. 2013). The structural model is based on Protein Data Bank accession 1H3*E* from *Thermus thermophilus* (Yaremchuk et al. 2002) and was visualized with Mol* (Sehnal et al. 2021).

A casual scan of the literature could yield radically different—but equally justifiable—conclusions about the robustness of genetic systems, depending on which corners of biology a reader happens to stumble into. On one hand, mutations that alter a single nucleotide can inactivate an entire gene and even produce lethal effects (Eyre-Walker and Keightley 2007), illustrating the fragility of many genetic systems. In other cases, organisms are astonishingly tolerant of major changes, such as genome-wide modifications to the genetic code (Mukai et al. 2017) or addition of entire genomes (Itaya et al. 2005; Tagwerker et al. 2012). This contrast is especially evident in molecular interactions between gene products. For example, a single-nucleotide substitution in a mitochondrial tRNA gene present in natural populations of the fruit fly *Drosophila simulans* has been shown to produce major incompatibilities when paired with a single amino-acid substitution in an interacting aminoacyl-tRNA synthetase (aaRS) enzyme from *Drosophila melanogaster* (Meiklejohn et al. 2013); and yet, aaRSs undergo widespread horizontal gene transfer (HGT) across disparate domains of life and functionally replace counterparts that are highly divergent in sequence (fig. 1; Woese et al. 2000). Similarly, a few amino-acid substitutions in interacting subunits within the mitochondrial NADH dehydrogenase complex of swordtail fishes (*Xiphophorus*) appear to be responsible for a lethal incompatibility (Moran et al. 2021); and yet, subunits within the mitochondrial ribosome, another mitonuclear (alphaproteobacterial-like) enzyme complex, have been entirely replaced by anciently divergent counterparts from plastid (cyanobacterial-like) or cytosolic (archaeal-like) ribosomes in some plant lineages (Adams et al. 2002). Such observations lead us to ask whether there are general principles to explain why certain systems are prone to rapid evolution of incompatibilities while others remain interchangeable even after billions of years of divergence.

We specifically selected the foregoing examples from the field of mitochondrial biology because the endosymbiotic history of eukaryotes may be especially valuable for disentangling the mechanisms that preserve interchangeability or lead to incompatibilities. The repeated merging of evolutionary lineages associated with the acquisition of mitochondria, plastids, and other bacterial endosymbionts creates redundancies between genetic systems and ample supply of material for HGT (which is also known as endosymbiotic or intracellular gene transfer in this context; Timmis et al. 2004; Sloan et al. 2018). Mitochondria and plastids retain their own genomes (albeit highly reduced ones) while also importing thousands of nuclear-encoded proteins. As a result, organellar functions depend on direct molecular interactions between gene products encoded in different genomes. For example, the major OXPHOS enzyme complexes responsible for cellular respiration are composed of both nuclear- and mitochondrial-encoded protein subunits (Rand et al. 2004; Burton et al. 2013). Even though they are found within the same cell, nuclear and cytoplasmic genomes can differ in key biological properties such as mode of inheritance, mutation rate, genome copy number, and expression level (Lynch et al. 2006; Smith and Keeling 2015; Forsythe et al. 2022). Such asymmetries can help test hypotheses regarding the evolutionary forces that contribute to genetic incompatibilities. The fact that nuclear and cytoplasmic genomes differ in so many ways also highlights one of the major challenges in identifying forces that shape the evolution of these incompatibilities. As we discuss at the end of this review, many apparently important factors are correlated such that progress will require new approaches to disentangle their effects.

Here, we review biological examples that illustrate the broad spectrum that ranges from incompatibility to interchangeability at the molecular level, pointing to four general principles that may explain where specific genes and functional pathways are placed along this spectrum.

## Genetic Incompatibilities Exposed by Hybridization and HGT

One of the central goals of evolutionary biology is to identify the genetic and molecular basis of reproductive barriers that lead diverging populations to eventually evolve into isolated species. Some common themes about the genomic architecture of reproductive isolation have emerged from analysis of natural and laboratory-generated hybrids, including the effect of inversions and other recombination suppressors (Schumer et al. 2018; Schluter and Rieseberg 2022) and the disproportionate role of sex chromosomes (Presgraves 2008, 2018).

Studies have also been increasingly successful in pinpointing examples of specific genes involved in postzygotic reproductive isolation in the form of so-called Bateson–Dobzhansky–Muller incompatibilities (BDMIs; table 1; Johnson 2010; Bozdag and Ono 2022). These incompatibilities represent a form of epistasis in which two or more variants function without detrimental effects in their respective genetic backgrounds but have harmful interactions when brought together by hybridization. The growing list of these “speciation genes” is enriched for certain functional categories. We have already noted examples of mitonuclear incompatibilities associated with direct physical interactions between mitochondrial gene products and imported nuclear-encoded proteins (Meiklejohn et al. 2013; Moran et al. 2021). These and similar examples have suggested that mitochondrial genes are frequent contributors to reproductive isolation and speciation (Burton and Barreto 2012; Hill 2016; Sloan et al. 2017; Postel and Touzet 2020; Bozdag and Ono 2022). Meanwhile, many of the nuclear genes that have been implicated in BDMIs are involved in various forms of genomic conflict and antagonistic coevolution, including centromere binding, transposable element activity, male sterility, testis-specific functions, and pathogen defense (Johnson 2010; Crespi and Nosil 2013; Sankararaman et al. 2014; Serrato-Capuchina and Matute 2018; Postel and Touzet 2020; Schluter and Rieseberg 2022). These recurring functional themes suggest that certain genes are more prone than others to developing incompatibilities.

**Table 1:** Examples of Molecular Genetic Incompatibilities Revealed by Hybridization Between Recently Diverged Lineage or by Gene Transfer (Either Natural or Experimental) Between More Distantly Related Taxa

Taxon	Description	Reference
***Hybrid incompatibilities***		
*Drosophila*	tRNA-aaRS mitonuclear interaction	Meiklejohn et al. (2013)
*Drosophila*	Lhr/Hmr heterochromatin interactions	Brideau et al. (2006)
*Xiphophorus*	OXPHOS complex I mitonuclear interaction	Moran et al. (2021)
*Mus*	PRDM9 and recombination hotspots	Mihola et al. (2009)
*Homo*	Testis-specific genes	Sankararaman et al. (2014)
*Saccharomyces*	AEP2/OLI1 mitonuclear interaction	Lee et al. (2008)
*Oryza*	S5 Proteases	Chen et al. (2008)
*Arabidopsis*	NLR immune receptor genes	Chae et al. (2014)
***Transfer incompatibilities***		
Tree of Life	Ribosomal proteins	Ciccarelli et al. (2006), Sorek et al. (2007)
Angiosperms	Plastid Clp protease	Abdel-Ghany et al. (2022)
Bacteria	ACCase	Wellner and Gophna (2008)
*Sinorhizobium*	BacA and plant nodulation coevolution	diCenzo et al. (2017)
Bacteria	DNA replication machinery	Jain et al. (1999), Sorek et al. (2007)
Bacteria	Elongation factor Tu	Kacar et al. (2017)
Plants/bacteria	Rubisco	Kanevski et al. (1999)
Bacteria	Dihydrofolate reductase	Bershtein et al. (2015)

Although hybridization and introgression studies have been highly informative in identifying genetic incompatibilities, they are inherently limited to recent histories of divergence because they depend on lineages that remain at least partially interfertile. The history of HGT between more anciently divergent lineages provides an alternative avenue to determine which genes preferentially build up incompatibilities and which remain highly interchangeable. Comparative studies have been valuable in identifying biological features associated with genes that are especially likely or unlikely to undergo HGT (Rivera et al. 1998; Sorek et al. 2007; Cohen et al. 2011; Creevey et al. 2011; Baltrus 2013; Nagies et al. 2020). Although most of this HGT work has focused on the gain of novel functions, HGT can also result in the replacement of homologous genes and existing functions (Koonin et al. 2001; Andam and Gogarten 2011; Creevey et al. 2011; Huang and Yue 2013; Nagies et al. 2020). Such examples of direct functional replacement via HGT are particularly relevant to the subject of this review because they inform our understanding of interchangeability.

Laboratory experiments have complemented comparative analyses of HGT by allowing for more controlled and systematic tests of gene transferability (table 1). In one classic study, Sorek et al. (2007) took advantage of the fact that early genome projects involved cloning shotgun gene libraries into *Escherichia coli*. The authors reasoned that gaps in genome assemblies that required closing by PCR could be used to identify genes that hindered *E. coli* growth and viability. More generally, heterologous expression and mutant rescue experiments in systems such as yeast and *E. coli* are commonly employed to test hypothetical gene functions that have been inferred from sequence homology (Minet et al. 1992; Sweasy and Loeb 1993; Perkins et al. 1999; Osborn and Miller 2007; Hamza et al. 2015). An implicit assumption of such approaches is that gene function is largely conserved across species (i.e., the orthology-function conjecture) and that it remains portable and interchangeable even when donor species come from radically different parts of the tree of life (Gabaldón and Koonin 2013). Conversely, failure of such experiments may reflect incompatibilities between a donor gene and the recipient species (Dick and Trumpower 1998; diCenzo et al. 2017).

More targeted studies have also directly tested for genetic incompatibilities by generating chimeric enzyme complexes with subunits derived from two different species or complexes with an altered mix of paralogous subunits (Kanevski et al. 1999; Kim et al. 2009; Lind et al. 2010; Kacar et al. 2017; Abdel-Ghany et al. 2022). In such experiments, it is also possible to make comparisons between the effects of introducing a single foreign (or ancestral) subunit versus replacing an entire multisubunit complex (Garcia et al. 2022). Likewise, cytoplasmic hybrid (cybrid) experiments, in which the nuclear genome of one species must function with the cytoplasmic genomes of another species, have documented incompatibilities associated with divergence between lineages (Kenyon and Moraes 1997; Schmitz-Linneweber et al. 2005). Overall, this array of comparative and experimental approaches has provided extensive examples of genetic incompatibility, which we will draw on in this review.

## Functional Interchangeability can be Maintained Across Ancient Timescales

The preceding section emphasized that genetic incompatibilities can have severe effects on molecular interactions and sometimes emerge over short timescales. However, comparisons across the tree of life have revealed contrasting examples, in which genes with core cellular functions have been exchanged across anciently divergent lineages and still retained their functions (table 2). In addition, laboratory experiments have been able to reconstitute complex molecular machinery with components from diverse donor species (McClintock et al. 2018). In this section, we overview some of the striking examples of interchangeability in molecular evolution.

**Table 2:** Examples of Interchangeability in Molecular Interactions Including Both Homologous and nonhomologous Replacement Events

Description	Reference
***Homologous replacement***	
Aminoacyl-tRNA synthetases (cellular tree of life)	Woese et al. (2000)
Mitochondrial ribosomal proteins (cellular tree of life)	Adams, et al. (2002)
Mitochondrial tRNAs (cellular tree of life)	Warren, et al. (2021)
Endosymbiont peptidoglycan biosynthesis (bacteria)	Husnik, et al. (2013)
Plastid GAPDH (cellular tree of life)	Keeling (2009)
in vitro reconstitution of dynein motor complex (metazoans)	McClintock et al. (2018)
Heteromeric and homomeric ACCase (cellular tree of life)	Konishi et al. (1996)
Mitochondrial DNA polymerase (cellular-viral tree of life)	Shutt and Gray (2006)
***Nonhomologous replacement***	
SUF sulfur mobilization system	Karnkowska et al. (2016)
Telomerase functions	Multiple (see text)
Cytochrome *c* maturation	Babbitt et al. (2015)
Siderophore biosynthesis	Bruns et al. (2018)
Classes I and II LysRS	Shaul et al. (2006)
Ribozyme and protein-only Rnase P	Lechner et al. (2015)
Fructose-6-phosphate aldolase (FBA)	Patron et al. (2004)
Superoxide dismutase	Sutherland et al. (2021)

As noted above, aaRS enzymes have undergone extensive HGT among all domains of life (Woese et al. 2000). Such patterns of interchangeability are also observed in tRNAs themselves. Mitochondria inherited tRNA genes from their bacterial progenitor, and some eukaryotes have retained a minimally complete set of these genes in the mitochondrial genome, but multiple lineages have lost many or all of them (Adams and Palmer 2003; Pett and Lavrov 2015; Salinas-Giegé et al. 2015). There are no known cases in which these tRNA genes have been transferred to the nucleus and targeted back to the mitochondria. Instead, mitochondrial tRNA gene loss has been accompanied by the import of the nuclear-encoded tRNAs that normally function in the cytosol, meaning bacterial-like tRNAs were replaced by their anciently divergent eukaryotic counterparts (Salinas-Giegé et al. 2015; Warren et al. 2021).

In other cases, the establishment and integration of endosymbiotic bacteria and organelles into eukaryotic host cells has depended on gene transfer to the nucleus. Surprisingly, however, many such transfers have not come directly from the endosymbiont but instead originated from other bacterial donors, suggesting replacement of machinery originally contributed by the endosymbiont. For example, peptidoglycan is one of the defining features of the bacterial cell wall, and peptidoglycan biosynthesis in some plastids and endosymbiotic bacteria is now controlled by nuclear genes. But phylogenetic analyses have traced these peptidoglycan biosynthesis genes to disparate bacterial lineages (Husnik et al. 2013; Sato and Takano 2017; Dowson et al. 2022), meaning that the native enzymes originally present in the endosymbionts have been functionally replaced by homologs from entirely different phyla. Such examples support the broader argument that establishment of endosymbiotic relationships may often involve a series of multiple relationships that leave genetic footprints (Larkum et al. 2007; Bennett and Moran 2015; Gray 2015).

The history of interchangeability in molecular evolution also extends to arguably the most fundamental processes of life—the replication and transcription of nucleic acids. For example, the DNA polymerase responsible for replication of mitochondrial DNA in animals, fungi, and other opisthokonts is not bacterial-like, contrary to what might be expected given the origins of mitochondria. Instead, the ancestral DNA polymerase has been functionally replaced by a viral-like polymerase; likewise, all eukaryotes appear to use viral-like machinery for helicase activity and transcription in their mitochondria (Shutt and Gray 2006), and the plastid genome of the cryptophyte *Rhodomonas salina* CCMP1319 was found to have acquired a gene encoding a putative DNA polymerase subunit from an unrelated bacterial lineage (Khan et al. 2007).

The foregoing examples highlight the widespread history of functional replacement between homologous genes across the tree of life (Creevey et al. 2011; Nagies et al. 2020). However, in even more extreme cases, native machinery can be replaced by a nonhomologous molecular system that plays a similar functional role (table 2). Such replacements are possible because many enzymes that catalyze the same reaction have evolved independently (e.g., the multiple structurally distinct superoxide dismutases distributed across the tree of life; Omelchenko et al. 2010; Sutherland et al. 2021).

A striking example of nonhomologous replacement involves the key roles of mitochondria in production of iron-sulfur clusters, which are so essential that parasitic eukaryotes that lose the ability to generate ATP through cellular respiration still retain mitochondrion-related organelles to perform this function (Tovar et al. 2003). The only known exception is the oxymonad *Monocercomonoides*, which appears to have lost mitochondria entirely. This loss was likely facilitated by HGT and the acquisition of a bacterial-like sulfur mobilization system (SUF) system as a nonhomologous alternative to produce iron-sulfur clusters (Karnkowska et al. 2016).

Above, we highlighted tRNAs and aaRSs as extreme cases of homologous functional replacement. However, lysine aaRSs have also been involved in nonhomologous replacement events. Lysine is the only aaRS with representatives in both of the (evolutionarily unrelated) Class I and Class II families, and these two alternative forms have undergone numerous functional replacement via HGT (Shaul et al. 2006). The enzyme responsible for processing the 5′ ends of tRNAs (RNase P) provides another example of interchangeability in tRNA metabolism, involving machinery that is functionally analogous but nonhomologous. The discovery that the catalytic activity of RNase P was conferred by an RNA and not a protein was a groundbreaking advance in the history of molecular biology, illustrating that RNAs can have enzymatic activity (ribozymes; Guerrier-Takada et al. 1983). As such, it came as a great surprise when it was later shown that RNase P activity in plant and animal mitochondria is mediated by a protein-only enzyme (Holzmann et al. 2008; Gobert et al. 2010). It has since become clear that both the ribozyme and protein-only versions of RNase P were ancestrally present in eukaryotes, and the subsequent history of differential gene retention and loss across lineages has determined which of these interchangeable versions now plays the functional role in tRNA processing (Lechner et al. 2015).

Repeated examples of interchangeable but nonhomologous machinery arise from the challenge of maintaining telomeres at the linear ends of chromosomes. Most eukaryotes extend their telomeres using the ribonucleoprotein telomerase complex, which relies on reverse transcription of a noncoding RNA to synthesize telomeric DNA (Podlevsky and Chen 2016). However, in several lineages, this function has been replaced by alternative mechanisms. For example, in *Drosophila*, telomeres are extended via a transposon-mediated system (Biessmann et al. 1990; Levis et al. 1993; Louis 2002) and a similar transition from telomerase-mediated to transposon-mediated telomere maintenance appears to have evolved independently multiple times in insects (Fujiwara et al. 2005; Mason et al. 2016). Mosquitos use yet another mechanism—one based on recombination—to extend telomeres (Roth et al. 1997). In addition, yeast lacking functional telomerase as well as certain human cancer lines have also been shown to perform recombination-mediated telomere elongation (Lundblad 2002; van Mourik et al. 2016; Zhang and Zou 2020), and *Myotis* bats also appear to use an alternative to the standard telomerase mechanism (Foley et al. 2018). Collectively, such examples illustrate the incredible extent to which evolution has produced alternative systems to solve the same problems and how such systems can sometimes be transferred across disparate branches in the tree of life.

## Genetic Principles that Determine Balance Between Incompatibility and Interchangeability

**Fig. 2.**
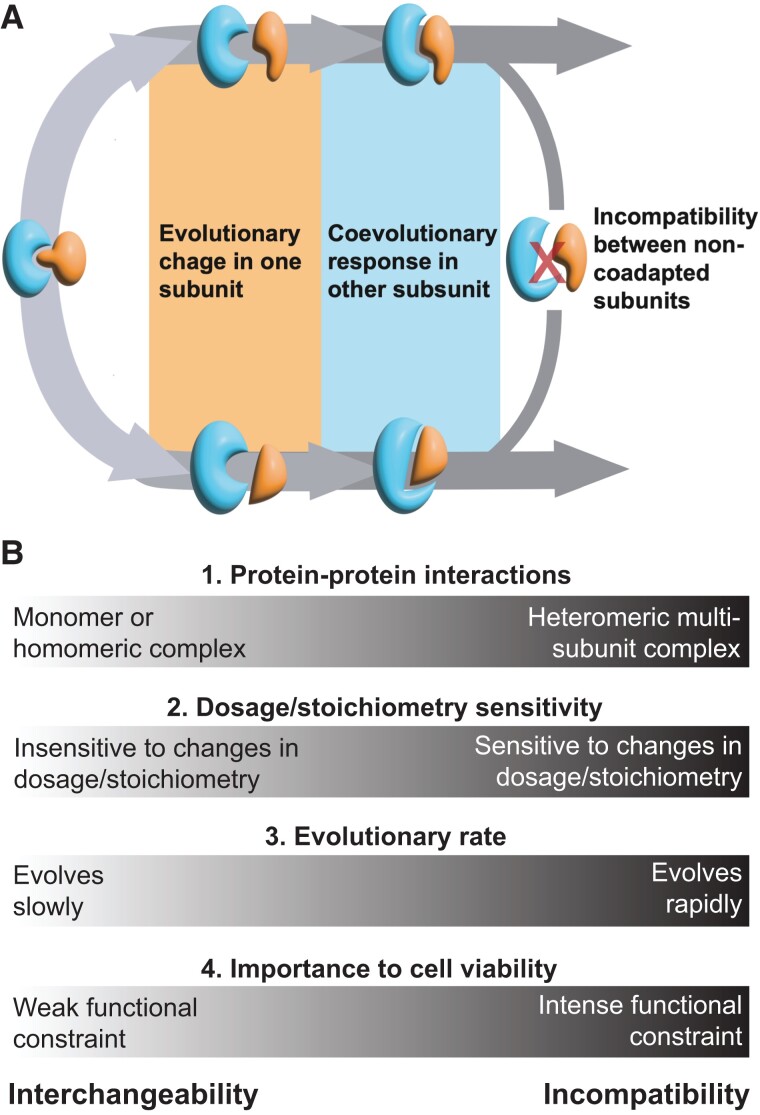
The origins of genetic incompatibilities: (*A*) Stylized representation of the coevolutionary process leading to incompatibilities between isolated evolutionary lineages. Interacting subunits (blue and orange) undergo evolutionary changes and coevolutionary responses, preserving functional interactions within a lineage but leading to incompatibilities between subunits when brought back together through hybridization or HGT. (*B*) Summary of genetic principles that may determine the balance between interchangeability and incompatibility in specific molecular systems.

How is it that some molecular systems rapidly evolve genetic incompatibilities while others remain interchangeable over deep evolutionary timescales? The answer to this question is undoubtedly complex and multifaceted, but below we point to four hypothesized genetic features that may contribute to where molecular systems fall on the incompatibility-interchangeability spectrum (fig. 2).

### Multisubunit Complexes and Extent of Protein–Protein Interactions

The “complexity hypothesis” and derivations thereof have suggested that interactions within stable multisubunit complexes as well as more transient protein interactions represent barriers to functional replacement (Jain et al. 1999). There is extensive evidence that interacting proteins coevolve (Clark and Aquadro 2010; de Juan et al. 2013; Forsythe et al. 2021; Neverov et al. 2021). Accordingly, disruption of these coevolved relationships through hybridization or HGT has the potential to produce incompatibilities (Swamy et al. 2021). This concept has been supported by a number of systematic and genome-wide tests, most of which have identified a negative relationship between a gene's number of protein–protein interactions and its propensity to undergo HGT (Jain et al. 1999; Sorek et al. 2007; Wellner et al. 2007; Lercher and Pál 2008; Creevey et al. 2011; Acar Kirit et al. 2020; Burch et al. 2022).

The ribosome is probably the most extensively documented example of a molecular system that is recalcitrant to functional replacement events. Because this massive, multisubunit enzyme complex appears to be largely resistant to HGT, ribosomal gene trees are generally viewed as representative of species relationships even at deep phylogenetic scales (Ciccarelli et al. 2006; Burch et al. 2022). In addition, the diverse range of interactions within the ribosome has facilitated more nuanced analyses. For example, ribosomal protein subunits with larger amounts of surface area in contact with ribosomal RNAs are more likely to produce incompatibilities (Sorek et al. 2007). Therefore, the intimacy and not just the quantity of molecular interactions is likely important in restricting interchangeability. Of course, the extent to which protein–protein interactions act as a barrier to interchangeability is not absolute. Even though the ribosome is often held up as the canonical example of a multisubunit complex with limited interchangeability, it is not entirely immune to HGT (Adams et al. 2002; Creevey et al. 2011). Likewise, subunits of the proteasome—another large multisubunit complex—exhibited a high degree of interchangeability in an experimental analysis of human-yeast orthologous proteins (Kachroo et al. 2015).

Another set of multisubunit complexes that have long been predicted to be a source of incompatibilities even over short timescales of divergence are the OXPHOS enzymes found in mitochondria (Rand et al. 2004; Burton and Barreto 2012; Hill 2016). This prediction arises from the following line of argument: (1) OXPHOS complexes are generally composed of both mitochondrial- and nuclear-encoded subunits, (2) mitochondrial genomes experience higher mutation rates and more rapid sequence evolution than in the nucleus in many eukaryotes, and (3) nuclear genes may experience selection for coevolutionary responses to changes in interacting mitochondrial genes, resulting in co-adapted mitonuclear genotypes that are sensitive to disruption by hybridization. Analyses of evolutionary rates and signatures of selection have found indirect evidence of coevolution between mitochondrial- and nuclear-encoded subunits in these complexes (Osada and Akashi 2012; Havird et al. 2015; Neverov et al. 2021), and a number of nuclear-encoded proteins that function in other aspects of mitochondrial biology have been implicated in BDMIs (table 1; Sloan et al. 2017; Bozdag and Ono 2022). However, specific examples of incompatibilities arising from interactions within OXPHOS complexes have remained somewhat limited (Burton 2022). Some of the most direct evidence with experimental support has come from examples of disrupted function in mitonuclear OXPHOS complexes in marine copepod hybrids (Ellison and Burton 2006; Harrison and Burton 2006) and the recently identified example of a lethal interaction within OXPHOS complex I in hybrid swordtail fish (Moran et al. 2021). As the tools to pinpoint such incompatibilities improve, it should become clear whether these examples are generalizable.

In some cases, the coevolved interactions among subunits within enzyme complexes may be discriminating enough to preclude any opportunity for functional replacement by horizontally transferred homologs. For example, the bacterial acetyl-CoA carboxylase (ACCase) enzyme consists of multiple subunits and catalyzes the conversion of acetyl-CoA to malonyl-CoA, a key early step in fatty acid biosynthesis (Salie and Thelen 2016). Experimentally transferring genes encoding one of the ACCase subunits from divergent bacterial donors into *E. coli*, which encodes its own native copies of these subunits, had negligible effects on measured growth rates; however, the reason for these limited fitness consequences appeared to be that the foreign subunits were too divergent to even assemble or interact with the native subunits at all (Wellner and Gophna 2008). Thus, there does not appear to be any potential to functionally replace the native gene with one of these foreign copies.

Protein–protein interactions and multisubunit complexes are thought to represent a barrier to functional replacement because preservation of coevolved interactions in these cases would necessitate simultaneous exchange and subsequent retention of multiple genes. Such multigene replacements may occur (Waller et al. 2006; Monier et al. 2009; Karnkowska et al. 2016). For example, they may be facilitated by the physical linkage or lack of recombination between functionally related genes, such as the introgression of mitochondrial or plastid genomes (Rieseberg and Soltis 1991; Toews and Brelsford 2012) or transfer of operons from bacterial or archaeal genomes (Omelchenko et al. 2003; Price et al. 2005). Nevertheless, multigene replacements are generally expected to be less probable than single-gene replacements (Keeling and Palmer 2008), which may explain some observed patterns of asymmetry in interchangeability. For example, plants typically have two distinct ACCase enzymes: (1) a typical eukaryotic multidomain homomeric ACCase that is encoded by a single gene and functions in the cytosol and (2) an endosymbiotically acquired bacterial-like heteromeric ACCase that consists of four different subunits and functions in the plastids. However, in multiple independent angiosperm lineages, the homomeric ACCase has been duplicated and now functions in both the cytosol and the plastids, in some cases leading to the loss of the heteromeric complex altogether (Konishi et al. 1996; Parker et al. 2014; Park et al. 2017; Williams et al. 2022). In contrast, the subunits of the heteromeric ACCase have not been found to be duplicated and retargeted to the cytosol. Similarly, mitochondria use one of two different systems to perform heme attachment as part of cytochrome *c* maturation. Many eukaryotes retain the ancestral bacterial-like enzyme, which consists of subunits encoded by six or more genes; however, this heteromeric complex has been lost and replaced by a single-gene system (the holocytochrome *c* synthase or HCCS) many times throughout eukaryotic evolution (Babbitt et al. 2015), a process which has likely included a history of HGT among eukaryotes (Allen et al. 2008). These recurring histories of replacement support the notion that transitions from multigene to single-gene systems are easier than the reverse process.

The history of functional replacement of mitochondrial aaRSs by their cytosolic counterparts also provides evidence for limitations imposed by multisubunit complexes in these replacement events. As described above, many lineages have lost some or all of their bacterial-like mitochondrial tRNA genes in favor of importing eukaryotic-like (nuclear) tRNAs from the cytosol (Salinas-Giegé et al. 2015). In such cases, it is common for the corresponding mitochondrial aaRSs to also be lost and replaced by retargeted cytosolic aaRSs, preserving the ancestral aaRS-tRNA charging relationship. However, the most notable and consistent exception to this appears to be the cytosolic phenylalanine aaRS. This enzyme is the only of the cytosolic aaRSs to be expressed as two different subunits, which likely hinders retargeting and functional replacement of its mitochondrial aaRS counterpart (Pett and Lavrov 2015; Warren et al. 2022). Therefore, in cases of mitochondrial tRNA-Phe loss, the native mitochondrial phenylalanine aaRS is retained and presumably must adapt to charge the newly imported cytosolic tRNA.

The idea that aaRSs could readily evolve to charge a novel tRNA substrate (see above) or undergo HGT across divergent lineages that span the tree of life (Woese et al. 2000) may seem surprising given the need for faithful aaRS-tRNA recognition in translation, but such evolutionary events may reinforce the hypothesized effects of molecular interactions in functional replacement. Accurate tRNA charging is generally achieved through the interaction between just two molecular components (the tRNA and the aaRS), and this interaction itself relies on a very small number of “identity elements” within the tRNA (Giegé et al. 1998). As such, the limited scope of molecular interactions may make aaRSs a relatively “modular” enzyme class and, thus, explain why they seem so amenable to HGT and functional replacement. The contrasting histories of plant and animal mitochondrial tRNAs offer some support for this interpretation. Plant mitochondrial tRNA genes have shown an extensive history of interchangeability and functional replacement (Small et al. 1999; Warren and Sloan 2020), which may indicate that the slow rate of sequence evolution in these genomes (Wolfe et al. 1987) has led to conserved tRNA sequences and structures that retain similarities with other translation systems. In contrast, animal mitochondrial tRNAs often have highly divergent sequences and noncanonical structures (Watanabe 2010; Salinas-Giegé et al. 2015; Warren and Sloan 2021), which may have resulted in highly coevolved and “locked in” relationships with their dedicated aaRSs. The very specific but limited basis of tRNA recognition may also help resolve the apparent paradox that we highlighted in Introduction. Whereas interchangeability may be maintained as long as the key tRNA identity elements are present, even small changes in sequence could lead to severe effects if they happen to disrupt this basis of recognition (Giegé et al. 1998; Meiklejohn et al. 2013).

The hypothesis that functional replacement is more likely to occur for proteins with limited molecular interactions is also supported by examples such as the extensive HGT in the peptidoglycan biosynthesis pathway for endosymbiotic bacteria/organelles (Husnik et al. 2013; Sato and Takano 2017; Dowson et al. 2022). The enzymes in this pathway catalyze individual reactions in series and do not assemble into large multisubunit complexes (Lovering et al. 2012). Likewise, the enzymes that act sequentially in the glycolysis pathway of eukaryotes are of endosymbiotic/bacterial origin and replaced the ancestral host machinery (Bártulos et al. 2018). More generally, the complexity hypothesis was initially conceived based on observations that “operational genes” (i.e., those involved in metabolic and housekeeping functions) are more likely to undergo HGT and less likely to be involved in extensive protein–protein interactions (Jain et al. 1999). As we have described in this section, subsequent studies in the last two decades have produced growing evidence that multisubunit complexes and protein–protein interactions can accelerate the accumulation of genetic incompatibilities and, thus, limit interchangeability.

### Sensitivity to Changes in Gene Dosage

Genes that are sensitive to changes in dosage (i.e., gene copy number and/or expression level) are often toxic when experimentally introduced into a host (Sorek et al. 2007; Acar Kirit et al. 2020). As such, dosage sensitivity may be a natural barrier to functional replacement because such replacements can entail a period of redundancy between native and foreign gene copies and, thus, changes in total expression level. Even in cases where direct homologous replacements have been engineered, expression levels can change with detrimental effects on fitness (Lind et al. 2010; Bershtein et al. 2015). Dosage sensitivity is a widespread biological phenomenon and has been linked to the concept of gene “balance” (Papp et al. 2003). Specifically, shifts in gene copy number or expression levels may disrupt molecular interactions that most occur at specific stoichiometric ratios. This phenomenon is thought to explain why whole-genome duplication (polyploidy) is often better tolerated than partial-genome duplication (aneuploidy) in many eukaryotes because the former generally maintains the same ratio of gene copy numbers, whereas the latter perturbs these ratios (Birchler and Veitia 2012).

One prediction arising from this dosage hypothesis is that genes that exhibit frequent functional replacement events can also readily be found in transitional states in which both copies are functional, implying that dosage effects of expressing two copies are not prohibitively costly. For example, as described above, the plastid heteromeric ACCase has been replaced in some taxa by importing the homomeric cytosolic ACCase, and species with both versions functioning in the plastid simultaneously have also been identified (Konishi et al. 1996; Parker et al. 2014; Park et al. 2017; Williams et al. 2022). Similarly, functional replacement of mitochondrial tRNAs by import of their cytosolic counterparts has been a common theme in eukaryotic evolution (Salinas-Giegé et al. 2015), and this replacement process appears to involve a phase of functional redundancy in which both types of tRNAs are simultaneously present in the mitochondria (Warren et al. 2021). More generally, this dosage hypothesis is supported by findings from genomic comparisons that genes that are preferentially maintained as single copy tend to be more resistant to HGT (Sorek et al. 2007).

Dosage effects may also apply to nonhomologous replacement. For example, it has been hypothesized that maintaining two distinct siderophore biosynthesis pathways (desferrioxamine or salinichelin) in *Salinispora* bacteria is harmful, explaining why the two pathways are never found in the same strain (Bruns et al. 2018). It is unclear whether such a cost is mediated by dosage effects, but it at least indicates any selective advantages from higher dosage and expression of two distinct pathways are insufficient to select for retention of both pathways. In this case, however, any barriers imposed by harmful redundancy have not (fully) prevented functional replacement, because multiple independent replacement events have been observed for these siderophore pathways.

Overall, these lines of evidence indicate that dosage sensitivity is a significant contributor to incompatibilities. As such, it is not just the nature of physical interactions that limits interchangeability but also the balance associated with levels of gene expression. However, the body of evidence in support of dosage sensitivity as a determinant of incompatibility versus interchangeability is arguably less extensive than for the other principles addressed in this review. Therefore, performing more systematic tests of this hypothesis will be important for further assessing its generality.

### Evolutionary Rate

Genes can evolve at remarkably different rates due to variation in the strength and efficacy of selection, the balance between positive and purifying selection, and differences in the underlying mutation rate (Bromham 2009). Because sequence divergence is expected to drive the accumulation of genetic incompatibilities (Presgraves 2010), genes with faster evolutionary rates may be less interchangeable. This hypothesis is supported by observations that the level of sequence divergence between taxa is negatively correlated with frequencies of HGT (Popa et al. 2011; Skippington and Ragan 2012; Williams et al. 2012; Slomka et al. 2020) and the ability of genes to functionally replace their homologs (Lind et al. 2010; Kacar et al. 2017). However, the overall level of sequence divergence confounds differences in divergence time with the effects of variation in evolutionary rate per se. Some studies have differentiated between these effects by comparing the transferability of orthologous genes from the same pairs of donor and recipient species (Kachroo et al. 2015; Burch et al. 2022). As such, divergence time is held constant so any differences in sequence divergence can be attributed to variation in evolutionary rates. These analyses found that genes with high rates of sequence divergence were indeed less amenable to HGT. They also hinted at the possibility that evolutionary rate effects may act synergistically with other factors, such as protein–protein interactions. For example, Burch et al. (2022) found that the negative relationship between evolutionary rate and HGT is stronger for genes involved in large numbers of protein–protein interactions. In addition, although Kachroo et al. (2015) surprisingly showed that most proteasome subunits were replaceable between humans and yeast despite the extensive protein–protein interactions within this complex, the main exceptions were the subunits of the *β* ring, which also exhibit faster rates of amino-acid sequence evolution than *α* subunits. Therefore, the combination of rapid evolution and protein–protein interactions may have an especially large effect.

In eukaryotes, cytonuclear interactions have been particularly useful in testing for rate effects because there are often systematic differences in evolutionary rates between the mitochondrial (or plastid) genome and the nucleus (Wolfe et al. 1987). For example, animal mitochondrial genomes often evolve substantially faster than the nuclear genome; thus, the accumulation of mitochondrial changes has been predicted to drive the coevolutionary process and select for compensatory responses in nuclear-encoded proteins that are targeted to the mitochondria and interact with mitochondrial-encoded gene products (Rand et al. 2004; Burton et al. 2013). Osada and Akashi (2012) tested for this predicted asymmetry using primate sequence data for proteins in the mitochondrial cytochrome *c* oxidase complex, showing that substitutions in mitochondrial-encoded subunits tended to precede substitutions at nearby sites in nuclear-encoded subunits. This apparent selection for compensatory or coevolutionary changes is one explanation for the observation that proteins targeted to the mitochondria often evolve faster than other nuclear-encoded proteins (Barreto and Burton 2013); however, more recent comparisons have not found that substitutions in mitochondrial- or plastid-encoded subunits are more likely to precede changes in the nuclear genome (Weng et al. 2016; Weaver et al. 2022). Taxa in which the rate of mitochondrial or plastid sequence evolution show large variation among closely related species have been especially useful for tests of these coevolutionary principles. Such tests have found strong correlations between evolutionary rates of cytoplasmic genomes and interacting nuclear-encoded proteins (Zhang et al. 2015; Weng et al. 2016; Havird et al. 2017; Yan et al. 2019; Forsythe et al. 2021).

Although accelerated rates and coevolutionary signatures from comparative-genomic studies are often assumed to be associated with a faster buildup of incompatibilities between divergent taxa, direct functional tests of this assumption have been rare. Nonetheless, some more targeted functional studies have engineered chimeric enzyme complexes or interaction networks by substituting in genes from donor species with varying levels of sequence divergence (Asai et al. 1999; Lind et al. 2010; Bershtein et al. 2015; Kacar et al. 2017). For example, Kanevski et al. (1999) engineered a rubisco enzyme complex in tobacco consisting of the native nuclear-encoded small subunit and a plastid-encoded large subunit that had been transferred from sunflower. This chimeric enzyme was able to successfully maintain partial rubisco functionality. However, the same was not true for attempts using a large subunit gene from a more distant (cyanobacterial) donor, supporting the expectation that the age of divergence between donor and recipient lineages contributes to accumulation of genetic incompatibilities. More recently, experiments used flowering plants that differed dramatically in their historical rates of sequence evolution for the plastid-encoded ClpP1 protein as donors to replace the native tobacco copy in another plastid–nuclear enzyme complex (the caseinolytic protease), finding that a history of accelerated sequence divergence hindered functional replacement (Abdel-Ghany et al. 2022). By using donors from the same genus (*Silene*), this experiment controlled for divergence time, isolating effects of evolutionary rate variation.

While cytonuclear interactions have been valuable in testing and teasing apart effects of evolutionary rate, such effects are also expected to pertain to nuclear–nuclear interactions. For example, the *PRDM9* gene is the best characterized example of a locus contributing to reproductive incompatibilities in mammals, and it undergoes unusually fast rates of sequence evolution (Mihola et al. 2009; Oliver et al. 2009). This gene is involved in determining hotspots for meiotic recombination by recognizing specific DNA sequence motifs, and its rapid evolution may reflect perpetual selection to recognize new motifs to counterbalance the predicted depletion of existing hotspots through recombinational mechanisms (Ponting 2011; Paigen and Petkov 2018). More generally, the antagonistic coevolution that is often associated with genomic conflict can often lead to rapid rates of sequence evolution, which may explain why genes involved in such conflict are often involved in BDMIs and reproductive isolation (Johnson 2010; Crespi and Nosil 2013; Sankararaman et al. 2014; Serrato-Capuchina and Matute 2018; Postel and Touzet 2020; Schluter and Rieseberg 2022). Therefore, differences in rates of sequence evolution appear to affect the balance between incompatibility and interchangeability in disparate evolutionary lineages.

### Overall Functional Importance

Perhaps the simplest and most intuitive hypothesis to explain observed variation in interchangeability is that the molecular systems that are especially important to cell viability and sensitive to disruption may be the most resistant to functional replacement. The rationale would be that the process of functional replacement inevitably involves some degree of perturbation to molecular systems, which would create more severe “fitness valleys” when they affect highly important genes. There is clear evidence that introduction of foreign genes and other forms of functional replacement can be disruptive through changes in protein homeostasis, increased cytotoxicity, and inefficient gene expression (Park and Zhang 2012; Baltrus 2013; Bershtein et al. 2015; Bedhomme et al. 2019). Even though subsequent evolution can lead to “amelioration” of such effects (Lawrence and Ochman 1997), the immediate harmful consequences may present too great a barrier to overcome for long-term functional replacement to occur, especially in the most constrained molecular systems.

Multiple observations support the hypothesis that functionally constrained genes are more resistant to replacement. For example, highly expressed genes are generally more conserved and have been shown to be less likely to undergo HGT (Park and Zhang 2012). In these cases, the barriers imposed by high expression may be associated with cytotoxic effects of inefficient translation and protein misfolding (Drummond et al. 2005; Zhang and Yang 2015). However, analysis of human-yeast orthologs identified the opposite pattern, as highly expressed genes were more likely to be replaceable in experimental complementation tests (Kachroo et al. 2015).

Many of the core components of molecular biology were present in the common ancestor of all extant cellular organisms and are near-universally conserved across the tree of life. Such systems are likely among the most important to cell function, and many of these appear to undergo lower rates of HGT and functional replacement than the rest of the genome (Jain et al. 1999; Fournier and Gogarten 2010; Koonin 2016). Indeed, the genealogical histories of proteins such as elongation factors G and Tu, RNA polymerase *β* chain, DNA polymerase III, signal recognition particle protein, and many ribosomal proteins closely resemble the structure of the tree of life with little history of reticulation (Brown et al. 2002).

A more direct measure of a gene's functional importance is the fitness effects associated with mutating it or knocking it out. At the extreme, many genes are considered essential because disrupting their function results in lethality (Glass et al. 2006; Wang et al. 2015). As noted above, proteins that have extensive molecular interactions are more resistant to functional replacement. Under what is known as the centrality-–lethality rule, these genes that encode highly interacting proteins are also more likely to be essential (Jeong et al. 2001; Hahn and Kern 2005; Wellner et al. 2007; Zotenko et al. 2008). The relatively rare cases where functional replacement of these essential molecular systems does occur may also be informative. For example, turnover of some core biochemical and molecular genetic machinery has been documented for mitochondria, plastids, and other bacterial endosymbionts (Hess and Börner 1999; Adams et al. 2002; Shutt and Gray 2006; Husnik et al. 2013; Gray 2015). In all these cases, the history of endosymbiosis has likely resulted in extreme bottlenecks and relaxation of selection pressures (McCutcheon and Moran 2012), which may have created a more permissive environment for functional replacement events that would have otherwise been too harmful. In the extreme, genetic degeneration in endosymbionts may be so severe that functional replacement events are not only tolerated but actually promoted by selection as a form of genetic “rescue” (Bennett and Moran 2015).

Overall, these lines of evidence all point to a role of functional importance in determining the balance between interchangeability and incompatibility.

## Open Questions and Future Directions

In this concluding section, we point to five areas where there may be opportunities to build on recent progress in our understanding of evolutionary forces that shape the process of functional replacement.

### Multifunctional Proteins: The Role of Pleiotropy in Evolution of Incompatibilities

One intuitive prediction is that genes that have multiple functions and affect multiple phenotypes (i.e., pleiotropy) have the potential to be involved in more genetic incompatibilities. However, this potential for incompatibilities may be mitigated by slower rates of evolution, as it has long been suspected that pleiotropy could act as a constraint on evolution (Fisher 1930; Orr 2000; Ngo et al. 2022). There is evidence that pleiotropic genes occupy central positions in protein–protein interaction networks (Promislow 2004). As we have discussed, such interactions are expected to directly affect a gene's interchangeability. In addition, genes with extensive protein–protein interactions also exhibit slower sequence evolution (Fraser 2005; Hahn and Kern 2005; Ngo et al. 2022) and more constrained gene expression (Lemos et al. 2004; Papakostas et al. 2014), which may also affect interchangeability. Likewise, pleiotropic genes appear to have more substantial phenotypic effects even when measured on a per-trait basis (Wang et al. 2010). Collectively, these patterns suggest that pleiotropy will affect the rate at which genetic incompatibilities arise. Indeed, modeling of gene regulatory networks has indicated that hybrid incompatibilities may most readily evolve under intermediate levels of pleiotropy (Tulchinsky et al. 2014). To our knowledge, however, the relationship between pleiotropy and a gene's amenability to functional replacement has not been experimentally tested. With the establishment of genotype–phenotype maps on genome-wide scales (Wagner and Zhang 2011), resources are increasingly available to investigate such effects.

### Decoupling Confounded Variables: Separating Correlated Genetic Features and the Phylogenetic Distribution of Donor Genes

Many of the genetic features we have discussed are not independent of each other, resulting in confounding effects that are difficult to disentangle. For example, as noted above, the functional importance of genes is associated with their degree of integration into protein–protein interaction networks (Jeong et al. 2001; Wellner et al. 2007; Zotenko et al. 2008). In other cases, features are negatively correlated (e.g., functional importance and evolutionary rate) and may mask each other's effects. Although some attempts have been made to distinguish the contributions of correlated variables (Cohen et al. 2011; Burch et al. 2022), separating such effects remains a pressing challenge and may require experimental manipulations to complement existing comparative and statistical approaches. For example, altering environmental conditions or modifying gene regulatory systems could be means to control gene expression levels during experimental transfers.

It is possible that adequately accounting for the effects of some features may also reveal additional principles that determine the balance between incompatibility and interchangeability. For example, we hypothesize that genes that are widespread across the tree of life would have a higher chance of functional replacement given the ample supply of potential donors. However, at face value, the available data do not support this hypothesis, as the most anciently conserved and widely distributed genes exhibit *less* HGT (Jain et al. 1999; Brown et al. 2002; Fournier and Gogarten 2010; Koonin 2016). Nevertheless, it is still possible that donor availability positively contributes to the probability of replacement once the confounded effects of functional importance are controlled for, suggesting a need for more targeted studies to address this question.

### Beyond *E. coli*: Expanding the Taxonomic Scope of Experimental Interchangeability Studies

Functional wet-lab analyses have provided a key complement to comparative-genomic and phylogenetic approaches in understanding the mechanisms of molecular incompatibility and interchangeability. Most of these groundbreaking studies have relied on the power of *E. coli* as a model system for high-throughput transgenic analyses to systematically screen the effects of gene transfer and functional replacement (Asai et al. 1999; Sorek et al. 2007; Bershtein et al. 2015; Kacar et al. 2017; Acar Kirit et al. 2020). However, there are many reasons to expect that the principles dictating the outcome of functional replacement may depend on the recipient genome and cellular environment. With the growing resources available for engineering the genomes of yeast and multicellular eukaryotes (Kachroo et al. 2015), there are exciting prospects to expand this field of functional studies beyond *E. coli*.

### Retracing the Steps: Use of Ancestral Protein Reconstructions in Functional Assays

A rapidly growing approach in the field of molecular evolution involves the use of phylogenetics to infer the sequence of ancestral protein-coding genes, which can then be synthesized and expressed (Hochberg and Thornton 2017). Such reconstructed ancestral proteins can then be used for functional assays both in vitro and in vivo (Smith et al. 2013; Kacar et al. 2017; Hochberg et al. 2020; Garcia et al. 2022; Kędzior et al. 2022). This approach addresses a fundamental limitation of conventional molecular incompatibility-interchangeability studies, which are typically restricted to analysis of extant proteins. Instead, inclusion of ancestral proteins presents the exciting opportunity to recreate the order and timing of the step-wise evolutionary process by which incompatibilities emerge and to determine how this evolutionary process plays out on complex epistatic fitness landscapes.

### Experimental Evolution: Capturing the Functional Replacement Process on Laboratory Timescales

An exciting recent development is the increasing use of experimentally evolved bacterial populations and whole-genome sequencing to track the effects of HGT across generations in the laboratory (Chu et al. 2018; Slomka et al. 2020; Woods et al. 2020; Power et al. 2021; Nguyen et al. 2022). These studies grow bacterial populations in the presence of various sources of donor DNA in the media or allow bacteria to evolve with other strains and potentially exchange DNA. As such, the outcomes of genetic exchange and functional replacements can be directly assessed under more realistic conditions of population growth and competition. Such approaches should create the opportunity to strategically manipulate donor and recipient genomes to further develop and test hypotheses about genetic features that affect the balance between incompatibility and interchangeability in molecular evolution.

## Supplementary Material

evac184_Supplementary_Data

## Data Availability

The data underlying this article are available in the article and in its online Supplementary material.
